# *N*‐n‐butyl haloperidol iodide mediates cardioprotection via regulating AMPK/FoxO1 signalling

**DOI:** 10.1111/jcmm.18049

**Published:** 2023-11-21

**Authors:** Binger Lu, Zhuomin Wu, Weiliang He, Zikai Feng, Jilin Liao, Bin Wang, Yanmei Zhang, Fenfei Gao, Ganggang Shi, Fuchun Zheng

**Affiliations:** ^1^ The First Affiliated Hospital Shantou University Medical College Shantou China; ^2^ Department of Pharmacology Shantou University Medical College Shantou China; ^3^ The Second Affiliated Hospital Shantou University Medical College Shantou China

**Keywords:** AMPK, cardioprotection, FoxO1, ischemia reperfusion, *N*‐n‐butyl haloperidol iodide, oxidative stress

## Abstract

Derangement of redox condition largely contributes to cardiac ischemia/reperfusion (I/R) injury. FoxO1 is a transcription factor which transcripts a series of antioxidants to antagonize I/R‐induced oxidative myocardial damage. *N*‐n‐butyl haloperidol iodide (F_2_) is a derivative derived from haloperidol structural modification with potent capacity of inhibiting oxidative stress. This investigation intends to validate whether cardio‐protection of F_2_ is dependent on FoxO1 using an in vivo mouse I/R model and if so, to further elucidate the molecular regulating mechanism. This study initially revealed that F_2_ preconditioning led to a profound reduction in I/R injury, which was accompanied by attenuated oxidative stress and upregulation of antioxidants (SOD2 and catalase), nuclear FoxO1 and phosphorylation of AMPK. Furthermore, inactivation of FoxO1 with AS1842856 abolished the cardio‐protective effect of F_2_. Importantly, we identified F_2_‐mediated nuclear accumulation of FoxO1 is dependent on AMPK, as blockage of AMPK with compound C induced nuclear exit of FoxO1. Collectively, our data uncover that F_2_ pretreatment exerts significant protection against post ischemic myocardial injury by its regulation of AMPK/FoxO1 pathway, which may provide a new avenue for treating ischemic disease.

## 1 INTRODUCTION

Ischemia/reperfusion (I/R) injury remains a major challenge which needs resolution to lower the occurrence of organ transplantation‐ or myocardial ischemia‐induced organ failure.^1^ Due to the heart being ischemic for prolonged periods prior to transplantation followed by reperfusion, I/R may occur during cardiac transplantation and cause myocardium injury.^2^ In heart transplantation, I/R damage could lead to acute and chronic rejection.^3^ Therefore, establishing a resolution to alleviate I/R injury would be beneficial to reduce the risk of cardiac graft rejection.

The components of heart I/R damage has been demonstrated to be multi‐faceted, which explains limited efficacious treatment.^4^ During the process of reperfusion, the surge of reactive oxygen species (ROS) has been reported to promote postischemic injury.^5^, ^6^ Forkhead box O1 (FoxO1) is a member of highly conserved protein family possessing common ‘Forkhead box’ DNA‐binding domain, which plays an essential role in energy metabolism, cell cycle progression, autophagy, apoptosis and ROS clearance.^7^, ^8^, ^9^ Especially, FoxO1 elevates the transcription level of a series of targeted genes encoding antioxidant proteins including SOD2 and catalase to mediates its protective effects against various stimuli‐induced oxidant stress.^10^, ^11^ Cardiac‐specific knock‐out of FoxO1 aggravates myocardial function and injury elicited by IR.^12^ However, the molecular signalling that modulates FoxO1 activity is still largely unknown. An attractive candidate may be AMP‐activated protein kinase (AMPK), the central energy sensor and widely reported cardioprotective factor.^13^, ^14^, ^15^ AMPK has been reported to phosphorylate FoxO1 and to enhance FoxO1 nuclear translocation.^16^, ^17^ Therefore, considering the significance of preservation of redox balance during heart I/R, it is important to elucidate the regulating mechanism of FoxO1 nuclear accumulation.

*N*‐n‐butyl haloperidol iodide (F_2_) is a compound derived from haloperidol (a classical and widely used antipsychotic agent), which confers maintained relaxing effect of coronary artery on F_2_ with no extrapyramidal reactions for its increased hydrophilicity. A series of our previous in vitro studies demonstrated that some mechanisms may be associated with the protective effects of F_2_, such as calcium channel blockage, reduced expression of early growth response gene‐1, modulation of protein kinase C activity as well as promotion of ROS scavenge.^18^, ^19^, ^20^, ^21^ In particular, an investigation from our group has indicated that F_2_ activates AMPK and subsequently suppresses ROS generation to mitigate hypoxia/reoxygenation (H/R)‐provoked oxidative damage in primary cultured heart endothelial cells.^22^ However, the mechanism by which AMPK exerts its antioxidative effects yet to be fully elucidated. Based on above‐mentioned evidence, this research is designed to decipher whether FoxO1 is vital in F_2_‐induced cardioprotection, and to further investigate the underlying regulating mechanism of FoxO1 signalling.

## 2 MATERIALS AND METHODS

### 2.1 Materials

F_2_ was synthesized by our laboratory.^19^ AMPK inhibitor Compound C (CC, Cat No. HY‐13418A) and FoxO1 inhibitor AS1842856 (AS, Cat No. HY‐100596) were purchased from MedChemExpress (Princeton, NJ, USA). cleaved caspase3 (Cat No. 9664), Bax (Cat No. 2772), FoxO1 (Cat No. 2880), AMPK (Cat No. 5831), P‐AMPK (Cat No. 2535), H3, catalase (Cat No. 14097), SOD2 (Cat No. 13141) and GAPDH (Cat No. 5174) were purchased from Cell Signalling Technology (Danvers, MA, USA), except for Bcl‐2 (Cat No. 26593‐1‐AP), caspase3 (Cat No. 19677‐1‐AP) and cTNT (Cat No. 15513‐1‐AP) from Proteintech (Rosemont, PA, USA). HRP‐conjugated (Cat No. BA1050, Cat No. BA1054) or fluorescent (Cat No. BA1032, Cat No. BA1101) secondary antibodies were purchased from Boster Biological Engineering (Wuhan, China). Dihydroethidium (DHE, Cat No.S0063) and 2‐(4‐Amidinophenyl)‐6‐indolecarbamidine (DAPI, Cat No. P0131) were purchased from Beyotime Biotechnology (Shanghai, China). Evans blue (EB, Cat No. E2129) and 2,3,5‐triphenyltetrazolium chloride (TTC, Cat No. 298964) were obtained from Sigma Aldrich (St. Louis, MO, USA). Super ECL reagent and collagenase type II were purchased from Thermo Fischer Scientific (Waltham, MA, USA).

F_2_ was dissolved in DMSO as a stock solution with a concentration of 50 mg/mL. After 1:100 dilution in normal saline, F_2_ (5 mg/kg) was intraperitoneally administered to test animals 24 h before myocardial I/R. DMSO administered to test animals was limited at 1% of all injections.

### 2.2 Animals

Adult C57BL/6 mice (male, 2–3 months) and neonatal mice (1–2 day) were used (Vital River Laboratory, Beijing). All experiments conducted to mouse were approved by the Institutional Animal Care and Use Committee of Shantou University Medical College and consistent with the *Guidelines for the Care and Use of Laboratory Animals* published by the National Institutes of Health. Animal suffering was minimized at the best of the current research.

### 2.3 Myocardial I/R protocol

Global I/R surgery was prepared as previously described.^23^ Following 1‐week adaption, one single dose of sodium pentobarbital (50 mg/kg) was intraperitoneally injected to mice to induce general anaesthesia. After complete anaesthesia, mice were intubated and connected to a rodent ventilator to ensure effective ventilation. Next, a midline sternotomy was applied to mice to expose heart. Subsequently, a slipknot was tied to the left anterior descending (LAD) coronary artery for 45 min to induce myocardial ischemia, followed by loosening the slipknot to induce reperfusion for 24 h. Successful preparation of this model was confirmed by the observation of cyanosis at apex of heart and elevated ST‐segment (Figure S1). Sham‐operated mice underwent the same procedure without LAD occlusion.

A preconditioning regimen was used to study the effect of F_2_ on cardiac I/R (Figure S2). The mice were divided into seven groups: (1) Sham; (2) I/R; (3) I/R + F_2_ (5 mg/kg); (4) I/R + AS (10 mg/kg); (5) I/R + AS (10 mg/kg) + F_2_ (5 mg/kg); (6) I/R + CC (10 mg/kg); (7) I/R + CC (10 mg/kg) + F_2_ (5 mg/kg). We previously demonstrated the safety of F_2_ on healthy mice.^24^ Thus, a F_2_ alone group was not included. In each experiment, both groups comprised six mice.

### 2.4 Infarct size assessment

Measurement of myocardial infarct size was carried out by TTC‐EB double staining. Briefly, after I/R, mice underwent the same anaesthesia procedure (see Section 2.3), followed by rapid excision of the hearts. Following re‐ligation of LAD coronary artery at the same occlusion place, the aorta of excised heart was cannulated with a catheter to perfuse 1% EB dye retrogradely to characterize the ischemic zone. Then, the hearts were frozen for 30 min at −80°C and cut into slices. These heart slices were incubated in 2% TTC for 30 min at 37°C to delineate the infarct zone. After fixation with 4% paraformaldehyde overnight, the slices were scanned and an ImageJ software (Media Cybernetics, Bethesda, MD, USA) was used to determine the infarction, risk and left ventricular (LV) areas. The infarct size was presented as infarct size (INF)/area at risk (AAR).

### 2.5 Evaluation of cardiac function

Echocardiography (ECG) was carried out to estimate the heart function by the Vevo 2100 imaging system (Visual Sonics Inc., Toronto, Ontario, Canada) as previously described.^23^ After mild anaesthesia using isoflurane, different groups of mice underwent transthoracic ECG to acquire high‐resolution echocardiographic images using 30‐MHz probe. The images were applied to measure a battery of indices of left ventricular function. These parameters were then used to calculate ejection fraction (EF) and fractional shortening (FS).

### 2.6 TUNEL assay

After anaesthesia, a separate group of mouse hearts underwent rapid excision and fixation by 4% paraformaldehyde solution for 12 h at 4°C. Following dehydration by a vacuum tissue processor (Thermo Fischer Scientific), hearts from different groups were embedded by paraffin and cut into 3–4 sections. To detect apoptosis, a commercial TUNEL assay kit (Cat No. G3250) from Promega (Madison, WI, USA) was employed following the instructions of the manufacturer. After the TUNEL and immunofluorescence staining for the myocardium marker cTnT, a LSM810 confocal microscope (Carl Zeiss AG, Oberkochen, Baden voorburg, Germany) was used to capture corresponding pictures. For quantification, myocardium apoptosis was presented as total nuclei (blue) count devided by TUNEL‐positive nuclei (green) count in every sharp field of each section in AAR region.

### 2.7 Measurement of myocardial enzyme release

The qualified serum samples were obtained by the centrifugation (3000 rpm) of collected blood specimens for 10 min. For the assessment of myocardial enzyme leakage, activity of serum creatine kinase (CK), lactate dehydrogenase (LDH) and MB isoenzyme of CK (CK‐MB) were assessed by commercial Bio‐sinew kits using Automatic Chemistry Analyzer (Toshiba Medical Systems Corporation, Otawara, Tochigi, Japan).

### 2.8 Cardiomyocytes isolation

Neonatal mice cardiomyocytes were isolated by enzymatic digestion from mice (1–2 day). Briefly, ventricle part of mice hearts was carefully collected and cut into pieces. After washing with PBS three times, the ventricular tissue was digested by collagenase type II (0.8 mg/mL) for 5 min at 37°C. The digested cell suspension was filtered and centrifugated for 6 min at 800 rpm. After removing supernatant, cell pellets were re‐suspended in M199 supplemented with 10% NBS and transferred to cell culture incubator for 30 min to discard adherent fibroblasts. Then, non‐adherent cardiomyocytes kept culturing for another 48 h.

### 2.9 Immunofluorescence

Detection of immunofluorescence was carried out as previously described.^22^ Cardiomyocytes cultured on coverslips were fixed and permeabilized with 0.2% Trition X‐100 for 30 min. Next, blocking buffer was employed to reduce background for 1 h at room temperature. Primary antibodies (AMPK and FoxO1) with 1:200 dilution were used to probe respective proteins at 4°C overnight. Cells were washed with PBS and incubated with secondary fluorescent FITC (green)‐and CY3 (red)‐conjugated antibodies. DAPI was used to stain the nuclei. A confocal LSM 810 (Carl Zeiss AG) microscopy was exploited to capture the images of distinct groups.

### 2.10 Western blot

Western blot was conducted as previously described.^24^ Equal heart protein samples (about 50 μg) were loaded to 10% SDS‐PAGE gels. The gels were then transferred to nitrocellulose membrane. The membranes were then blocked and incubated with primary antibodies (described in Section 2.1) at 4°C overnight. Following incubation with HRP‐conjugated secondary antibodies, the immunoblots were probed by super ECL reagent. The signals of corresponding bands were detected using a Bio‐Rad imaging system and quantified using a Gel‐Pro software (Media Cybernetics, USA). GAPDH was defined as the loading control to compare band intensity of different groups.

### 2.11 Co‐immunoprecipitation

Co‐immunoprecipitation analysis was conducted to detect AMPK‐FoxO1 interaction. In brief, heart tissue lysates were acquired using NP‐40 lysis buffer (Cat No. P0013F, Beyotime). Following protein concentration determination, equal amount of samples (about 500 μg) were incubated with 1.5 μg of primary antibodies at 4°C overnight on a rocker. Protein A/G magnetic beads (Cat No. HY‐K0202, MedChemExpress) were then used to precipitate antibody‐bound proteins. After 4 h precipitation at room temperature, the beads were separated by a magnetic stand (Thermo Fischer Scientific) and washed with ice‐cold PBST solution (0.1% Tween‐20 in PBS). After elution in SDS loading buffer in boiling water for 5 min, eluted proteins were further analysed by western blot.

### 2.12 Real‐time PCR (RT‐PCR)

RT‐PCR was performed as previously described.^24^ Total mouse heart RNA was isolated using Trizol (Cat No. 9108, Takara, Kyoto, Japan) and reversely transcribed to cDNA by employing a reverse transcription system (Cat No. RR037A, Takara). SYBR Green master mix (Cat No. Q121‐1, Vazyme Biotech, Nanjing, China) was exploited to prepare the reaction system to perform RT‐PCR experiment using a LightCycler480 system (Roche Inc., Basel, Switzerland). Relative expression of targeted genes was normalized over 18S RNA. All primer pairs were derived from a previously published document.^25^

### 2.13 Nuclear and cytosolic protein isolation

Isolation of nuclear and cytosolic fraction was developed based on a method previously reported by Calvert et al.^26^ Briefly, mouse heart samples were homogenized in buffer A with protease inhibitor cocktail, EDTA, Tris–HCl, sucrose and then centrifugated at 1000 g for 10 min. Cell pellets were washed and underwent resuspension in RIPA lysis buffer. Following centrifugation, supernatants were collected as the nuclear fractions. In contrast, followed by the first round of centrifugation, the supernatants were subjected to a second round of 100,000 g centrifugation for 1 h to obtain the cytosolic fractions.

### 2.14 Statistics

All data were presented as mean ± SEM. Comparisons between two groups were assessed by two‐sided unpaired Student's *t*‐test in Prism 9 (GraphPad Software, La Jolla, CA, USA). Data groups were compared using one‐way anova with Dunnett post hoc analysis. *p* < 0.05 was considered significant.

## 3 RESULTS

### 3.1 Pretreatment of F_2_ limits cardiac I/R injury and improves heart function

**FIGURE 1:**
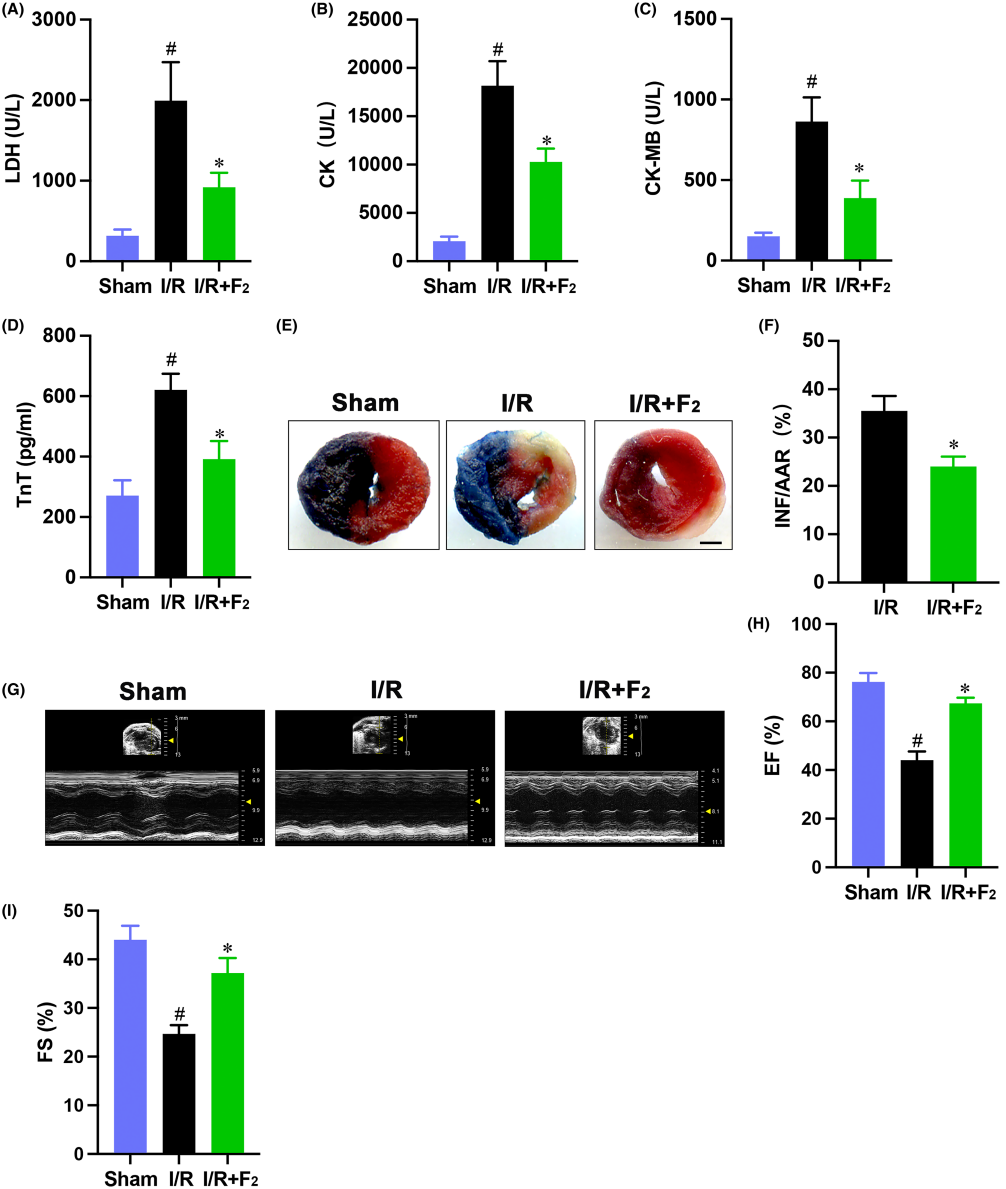
F_2_ protects mouse hearts against myocardial ischemic damage. (A–D) Effects of F_2_ on serum LDH, CK, CK‐MB and TnT concentration (*n* = 6). (E, F) Representative images and quantification analysis for infarction in hearts, scale bar = 1 mm (*n* = 5). (G–I) Representative echocardiograms and quantification analysis for EF and FS (*n* = 5). Data represent mean ± SEM. ^#^ *p* < 0.05 versus the Sham group; *p < 0.05 versus the I/R group.

Initial experiments were performed to test whether F_2_ pretreatment could ameliorate cardiac injury following I/R. F_2_ were intraperitoneally administered to mice 24 h before I/R. F_2_ significantly reduced the INF:AAR ratio (Figure 1E,F), although AAR per LV were comparable (data not shown). Of note, F_2_‐treated hearts were tolerant against myocardial I/R‐induced necrosis, as indicated by deceased leakage of myocardial enzymes including LDH, CK and CK‐MB (Figure 1A–D). Furthermore, cardiac function almost restored to baseline in F_2_‐treated mice, as evidenced by the apparent preservation in EF and FS (Figure 1G–I; Table S1). Therefore, these data demonstrate that F_2_ is a potent cardioprotective compound.

### 3.2 Preconditioning of F_2_ alleviates the extent of apoptosis and inflammation

**FIGURE 2:**
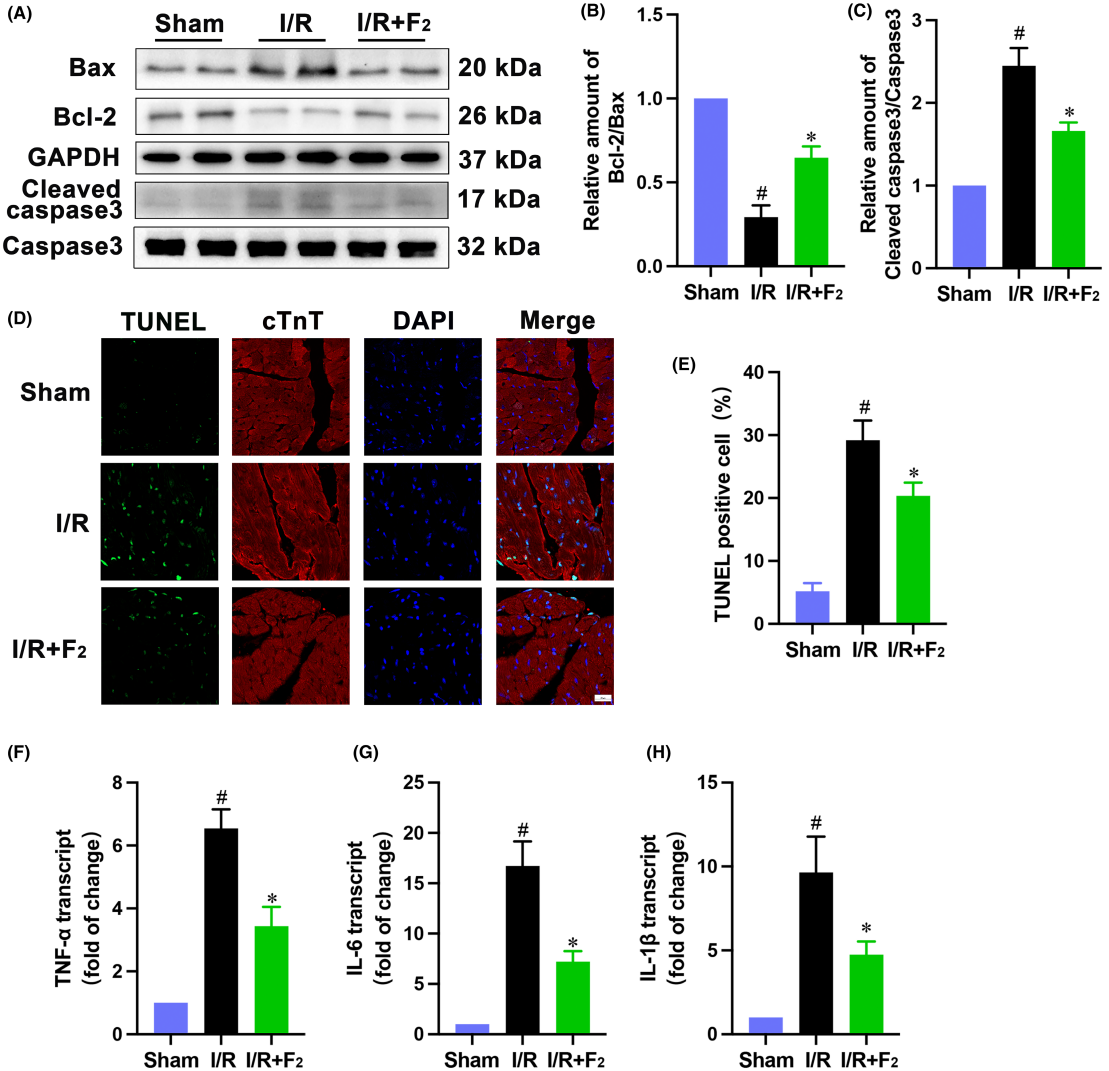
F_2_ ameliorates I/R‐triggered apoptosis and inflammation. (A–C) Western blot was employed to determine the expression of cleaved caspase 3, caspase3, Bax and Bcl‐2 (*n* = 6); (D, E) Apoptotic cardiac myocytes were assessed using the TUNEL assay, scale bar = 20 μm (*n* = 6); (F–H) RT‐PCR was exploited to detect the extent of inflammation (*n* = 6). Data represent mean ± SEM. ^#^ *p* < 0.05 versus the Sham group; *p < 0.05 versus the I/R group.

Because apoptosis substantially contributes to the cardiomyocytes death in ischemic heart disorder,^27^, ^28^, ^29^ we evaluated whether F_2_ preconditioning could reduce this form of cell death. Western blot analysis showed that myocardial apoptosis was evidently restricted by F_2_ preconditioning. This was as evidenced by the prevention of increase in Bax, cleaved caspase‐3 expression, and reduction in Bcl‐2 expression (Figure 2A–C), which was also supported by the decreased TUNEL positive myocyte counts (Figure 2D,E). Given that inflammation exerts a key role in postischemic injury,^30^, ^31^ we next estimated the potential effects of F_2_ on inflammation responses. The transcription of pro‐inflammatory cytokines such as IL‐6, IL‐1β and TNF‐α was markedly amplified during I/R but not in F_2_‐preconditioned hearts (Figure 2F–H). These results suggest that repression of inflammation and apoptosis may be responsible, at least partially, for cardioprotection conferred by F_2_.

### 3.3 F_2_ stimulates AMPK phosphorylation and increases nuclear FoxO1 import and the expression of SOD2 and catalase

**FIGURE 3:**
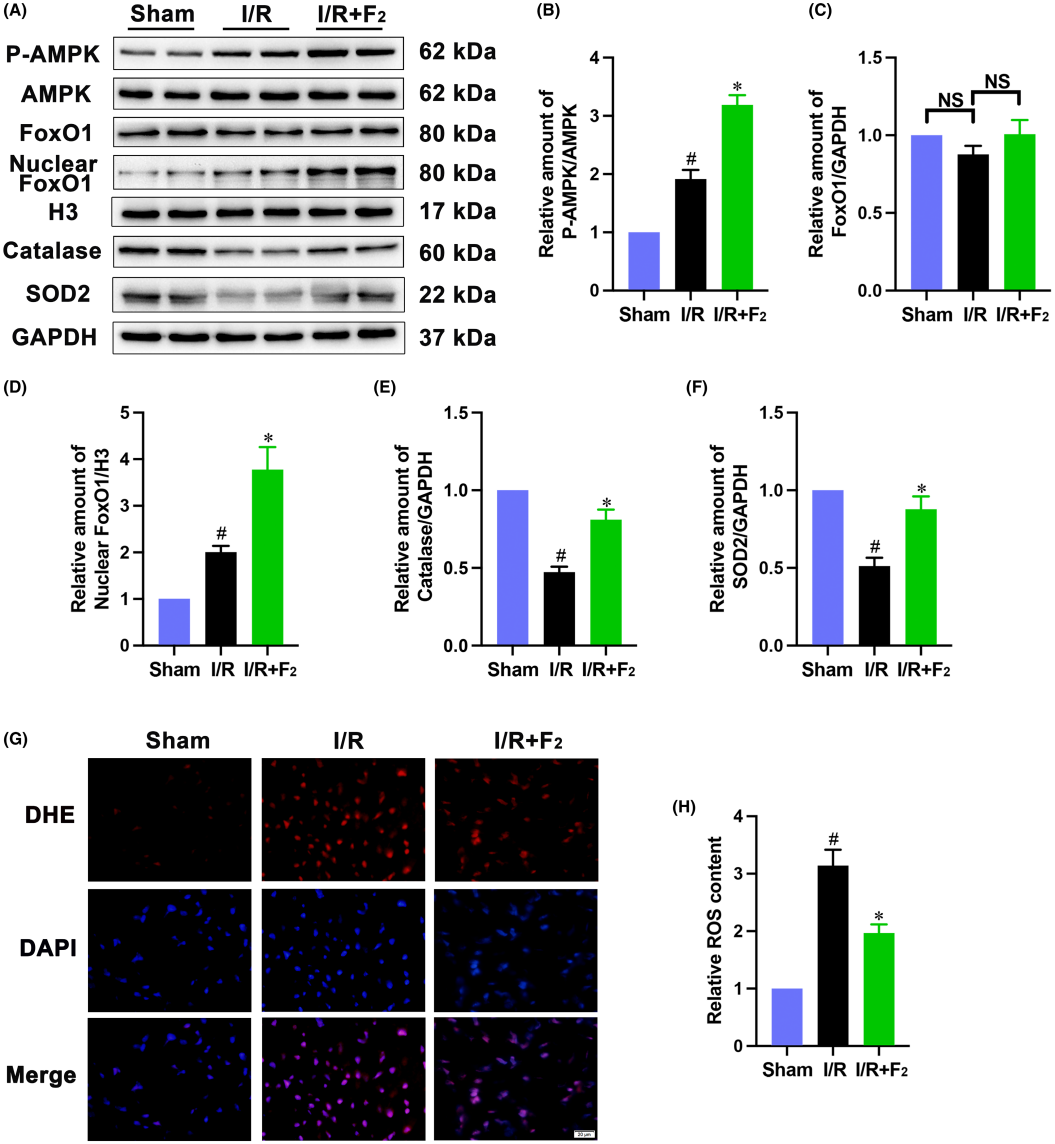
F_2_ enhances AMPK phosphorylation and nuclear FoxO1 accumulation in the I/R‐challenged heart. (A–F) Expression of nuclear FoxO1, FoxO1, AMPK, phospho‐AMPK, Catalase and SOD2 was examined by western blot (*n* = 6). (G, H) ROS generation was detected by DHE staining, scale bar = 20 μm (*n* = 6). Data represent mean ± SEM. ^#^ *p* < 0.05 versus the Sham group; *p < 0.05 versus the I/R group. H3 indicates the histone H3, a nuclear marker.

Next, we tested whether F_2_ might maintain redox homeostasis through AMPK and FoxO1 signals. As expected, I/R induced an adaptive increase in phosphorylation of AMPK and nuclear import of FoxO1, and this induction was augmented in F_2_‐preconditioned hearts (Figure 3A,B,D). Furthermore, I/R resulted in downregulation of SOD2 and catalase expression, which was corrected by F_2_ (Figure 3E,F). Intriguingly, I/R caused a moderate but not significant decrease of total FoxO1 expression, whereas F_2_ could mildly upregulate FoxO1 expression (Figure 3C). F_2_ also relieved I/R‐stimulated oxidant stress, as detected by DHE staining (Figure 3G,H). These results indicate that F2 might attenuate oxidative stress by stimulating AMPK/FoxO1 pathway.

### 3.4 FoxO1 is involved in the cardioprotection of F_2_

**FIGURE 4:**
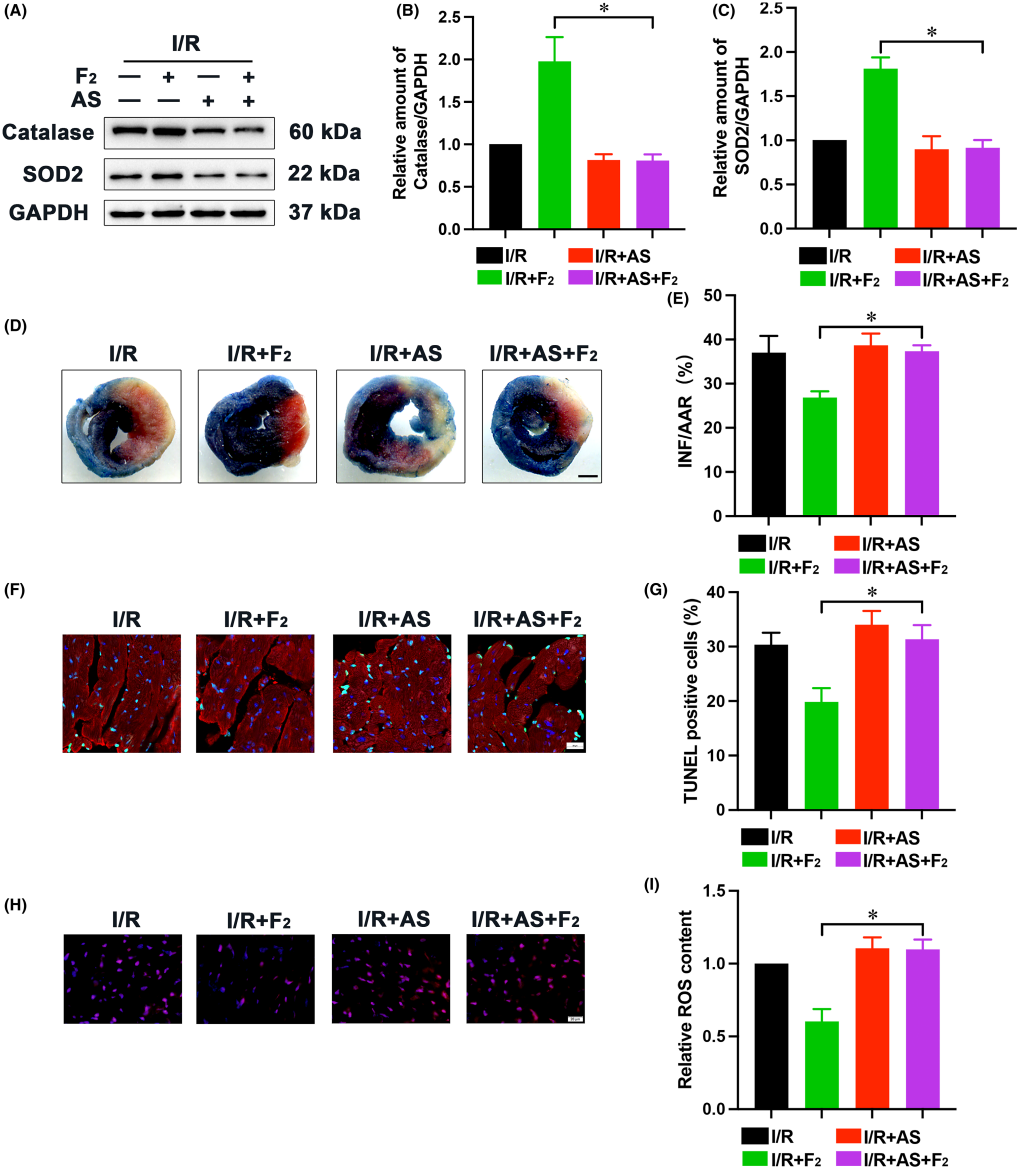
Involvement of FoxO1 in F_2_‐mediated cardioprotection. (A–C) Expression of antioxidant factors (Catalase and SOD2) were assessed by western blot (*n* = 6); (D, E) Representative photographs and quantification analysis for infarction in hearts, scale bar = 1 mm (*n* = 5); (F, G) The extent of cardiomyocytes apoptosis was determined by TUNEL staining, scale bar = 20 μm (*n* = 6); (H, I) ROS production was detected by DHE staining, scale bar = 20 μm (*n* = 6). Data represent mean ± SEM. *p < 0.05 versus the I/R + F_2_ group.

To determine whether FoxO1 is essential for the repressive effects of F_2_ on cardiac I/R lesion, FoxO1‐specific inhibitor AS was used. For these experiments, AS (10 mg/kg) was intraperitoneally administered to mice at 48 h and 24 h before ischemia. AS blunted the F_2_‐mediated increment of SOD2 and catalase expression in ischemic myocardium (Figure 4A–C). On the contrary, AS had no significant influence on the INF:AAR ratio in I/R‐challenged hearts and nearly abrogated the depressive effects of F_2_ on myocardial infarct size (Figure 4D,E). Additionally, F_2_‐induced reduction of TUNEL‐positive nuclei and ROS production was also abolished by AS (Figure 4F–I). Thus, F_2_‐mediated cardioprotection is correlated with FoxO1‐evoked antioxidant signalling pathway.

### 3.5 AMPK mediates F_2_ stimulation of FoxO1 signalling

**FIGURE 5:**
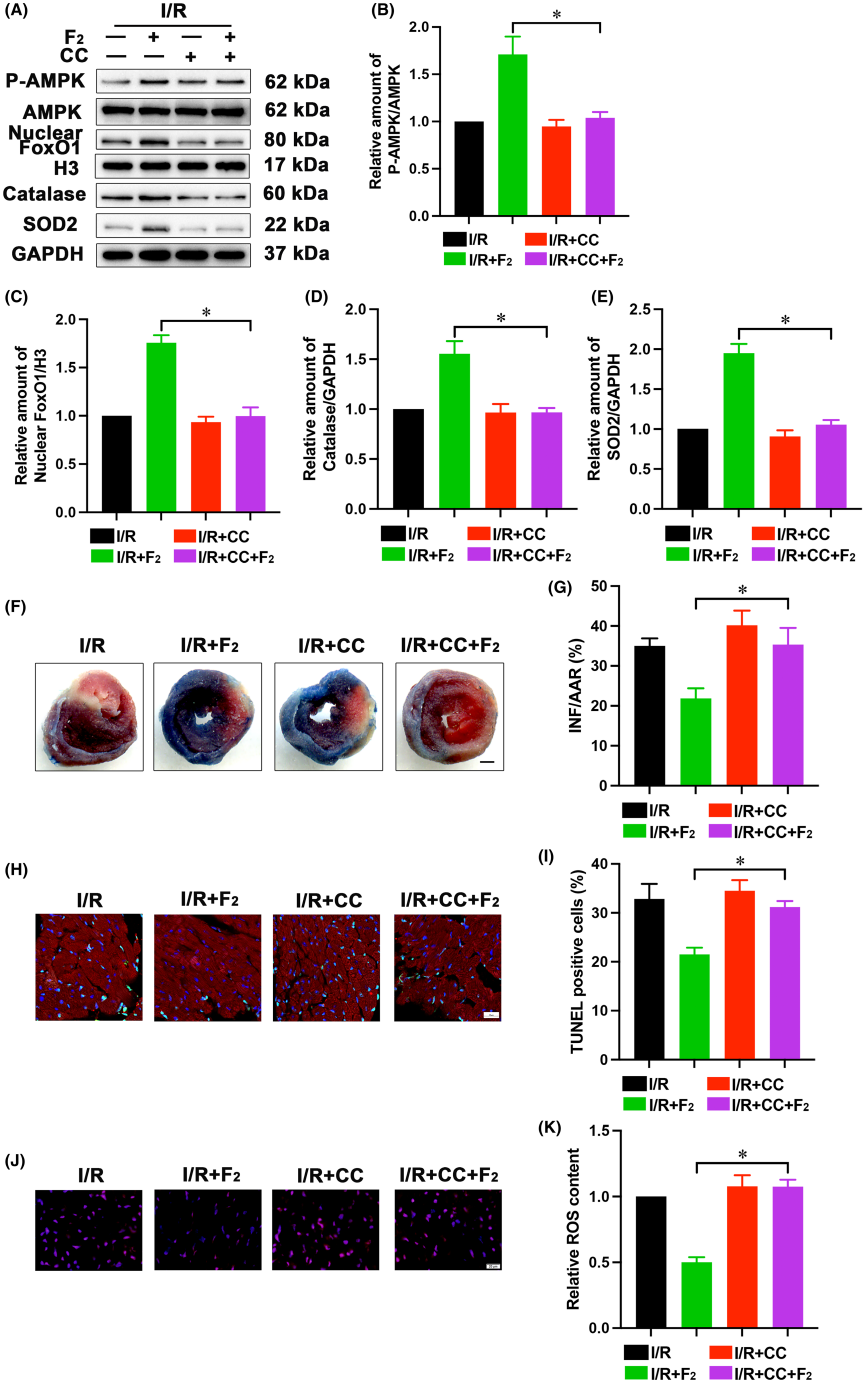
Blockade of AMPK relieves effects of F_2_ on FoxO1 activation in the ischemic heart. (A–E) Western blot was employed to examine expression of nuclear FoxO1, FoxO1, AMPK, phospho‐AMPK, Catalase and SOD2 (*n* = 6); (F, G) Representative pictures and quantification analysis for infarction, scale bar = 1 mm (*n* = 5); (H, I) TUNEL staining was employed to evaluate apoptotic cardiac cells, scale bar = 20 μm (*n* = 6); (J, K) DHE staining was utilized to estimate ROS production, scale bar = 20 μm (*n* = 6). Data represent mean ± SEM. *p < 0.05 versus the I/R + F_2_ group.

We subsequently aimed at determining whether F2‐induced activation of FoxO1 signalling was associated with AMPK. In this context, AMPK inhibitor CC (10 mg/kg) was also intraperitoneally administered to mice at 48 h and 24 h prior to I/R. AMPK Inhibition with CC abrogated the F_2_‐mediated nuclear FoxO1 accumulation and upregulation of SOD2 and catalase in I/R‐exposed hearts (Figure 5A–E). Although CC did not impact on myocardial infarct size, TUNEL‐positive cardiac cells and ROS generation in vehicle‐treated mice, CC almost abolished the suppressive effects of F_2_ on cardiac infarct area, apoptosis and oxidative stress after I/R (Figure 5F–K). These findings suggest that AMPK is an upstream mediator for F_2_‐induced activation of FoxO1 antioxidative signalling cascade.

### 3.6 AMPK interacts with FoxO1

**FIGURE 6:**
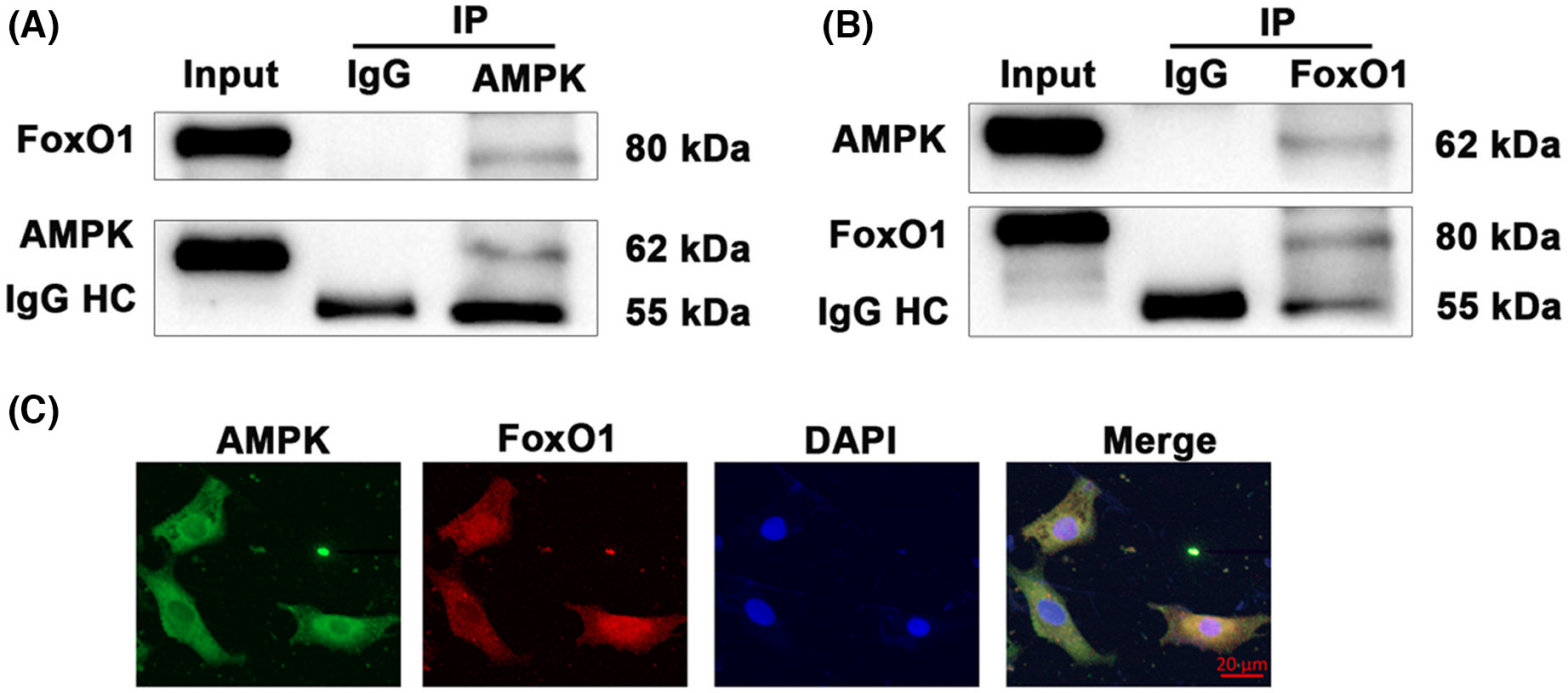
The interaction between AMPK and FoxO1. (A) After immunoprecipitation with anti‐AMPK antibody or rabbit IgG, western blot was employed to examine the expression of AMPK and FoxO1; (B) After immunoprecipitation with anti‐FoxO1 antibody or rabbit IgG, western blot was employed to examine expression of AMPK and FoxO1; (C) Representative immunofluorescent images showing the co‐localization of AMPK (green) and FoxO1 (red) in cardiomyocytes, scale bar = 20 μm. HC indicates the non‐specific IgG heavy chain bands.

Given our findings and the close relationship between AMPK and FoxO1 signalling, AMPK‐FoxO1 interaction was confirmed by co‐immunoprecipitation and immunofluorescence in cardiomyocytes. As shown in Figure 6A,B, FoxO1 was detected in AMPK immunoprecipitates and AMPK also co‐immunoprecipitated with FoxO1, suggesting a physical interaction between these two proteins. Moreover, consistent with the results of co‐immunoprecipitation, substantial colocalization of AMPK and FoxO1 were observed in cardiomyocytes using immunofluorescence staining (Figure 6C).

## 4 DISCUSSION

The current research firstly showed that F_2_ preconditioning potently protects mouse myocardium from I/R injury. F_2_ pretreatment also led to remarkable reduction in inflammation and apoptosis, which was supported by the markedly reduced expression of pro‐inflammatory and pro‐apoptotic factors. However, suppression of I/R damage by F_2_ was reversed by FoxO1 blockage with AS, suggesting that F_2_‐induced protection against myocardial I/R insult was FoxO1‐dependent. Furthermore, AMPK inhibition with CC abolished F_2_‐induced nuclear FoxO1 accumulation and the subsequent upregulation of antioxidant factors including SOD2 and catalase, resulting in the loss of F_2_ cardioprotective effects. Therefore, we conclude that pharmacological F_2_ pretreatment attenuates cardiac I/R injury via activating AMPK‐FoxO1 axis.

Under physiological conditions, a small proportion of ROS generated is rapidly scavenged by internal antioxidative systems.^32^ However, these systems are severely disrupted during I/R, which contributes to excessive ROS production and leads to the initial myocardial reperfusion injury.^33^ Thus, enhancing the activity of antioxidative enzymes may be a good strategy to decrease the oxidative stress‐elicited reperfusion damage.^34^, ^35^ Our previous work showed that F_2_ protected against H/R model‐evoked oxidative injury in CMECs and myocytes.^21^, ^22^ In agreement with these results, we found that the in vivo I/R‐induced oxidative injury was also significantly alleviated by F_2_ pretreatment. This alleviation was also associated with the upregulation of nuclear FoxO1 translocation and its downstream antioxidants SOD2 and catalase. Moreover, FoxO1 blockage restricted the inhibitory effects of F_2_ on myocardial injury. Thus, myocardial protection of F_2_ is mediated by its activation of FoxO1‐dependent antioxidative signalling.

As a major energy metabolic regulator, AMPK has been extensively documented to confer benefits on myocardium during I/R.^14^, ^15^ We previously reported that F_2_ promoted the phosphorylation of AMPK in endothelial cells,^22^ whereas this is the first study reporting AMPK activation by F_2_ in vivo. Importantly, we observed that the cardio‐protection of F_2_ was blocked by the administration of AMPK selective inhibitor, suggesting that activating AMPK is indispensable for its resistant effects against I/R injury.

We also found that AMPK was implicated in F_2_‐induced FoxO1 activation, which was evidenced by increased nuclear exit of FoxO1 in case of the F_2_ pretreatment group by AMPK inhibition. This observation is in parallel with the results of several studies.^16^, ^36^ Furthermore, suppression of AMPK likewise reversed the subsequent upregulation of antioxidant proteins. Importantly, the current study firstly reports that AMPK interacts with FoxO1 in cardiomyocytes. Collectively, these data indicate that F_2_ counteracts myocardial I/R damage via AMPK‐FoxO1 signalling. Notably, an important unanswered question exists with regard to the precise mechanisms by which AMPK activation activates FoxO1, which may relate to the phosphorylation of Thr^649^ residue and will be investigated in vitro cardiomyocytes H/R model in our further studies using genetical methods such as small interfering RNA other than pharmacological inhibition.

Another question worthy of explanation is that there are some discrepancies existing in our work. Here, we showed that, during I/R, phosphorylated AMPK and nuclear FoxO1 accumulation were enhanced, whereas the expression of downstream antioxidants including SOD2 and catalase was decreased. These seemingly contradictory data may be deciphered by a documented landmark investigation which showed that myocardial I/R activates Hippo pathway and induced the reduced accumulated Yes‐associated protein (YAP, a nuclear co‐factor of FoxO1) in the nucleus, resulting in the downregulation of FoxO1‐targeting gene transcription, despite elevated nuclear translocation of FoxO1.^37^ Whether F_2_ regulates the Hippo‐YAP signalling axis needs investigation in future studies.

**FIGURE 7:**
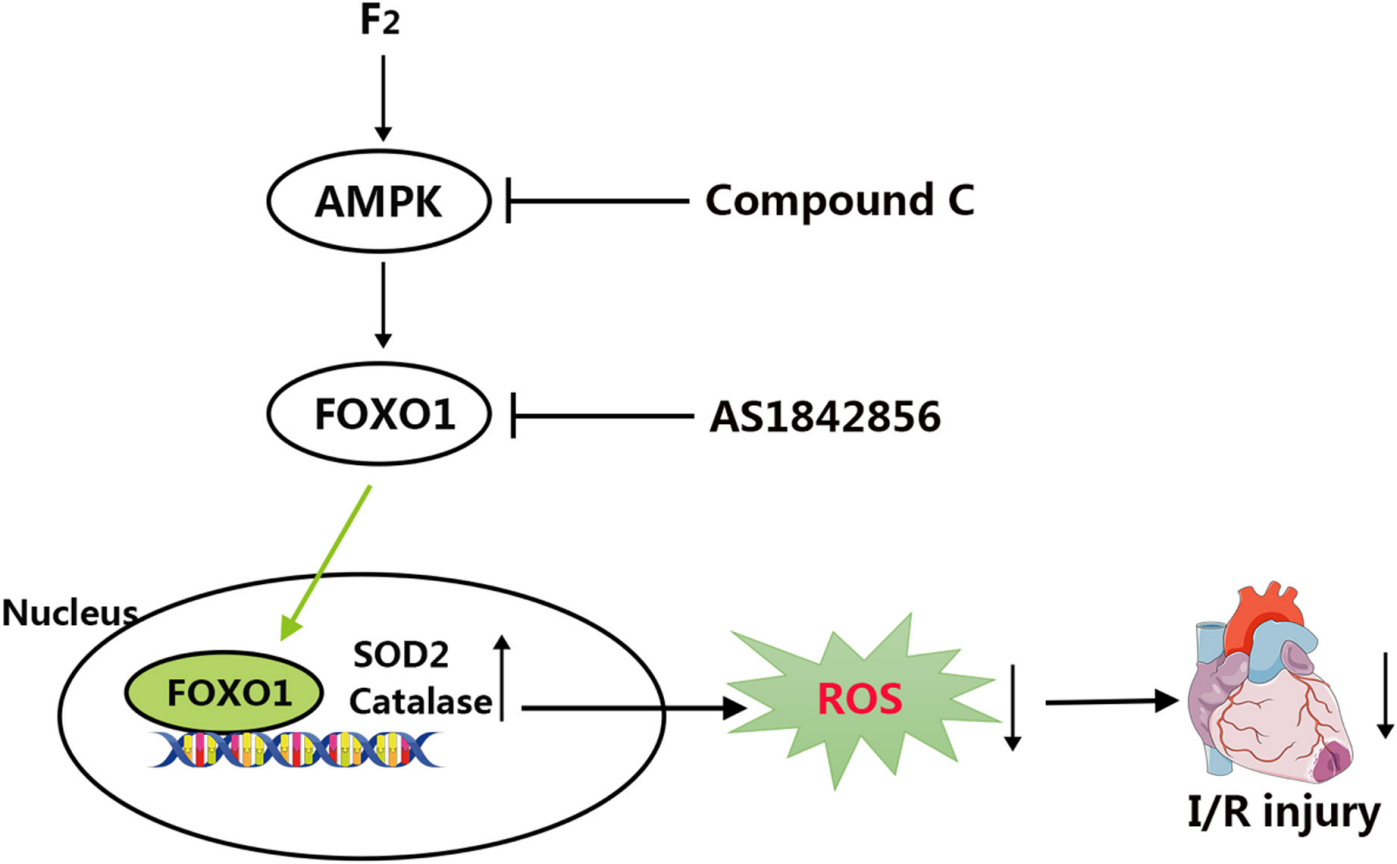
Scheme illustrating the current investigation. We propose that F_2_ increases FoxO1 nuclear translocation through activating AMPK, thereby promoting the transcription of antioxidant proteins, including SOD2 and Catalase. These increased levels of antioxidants negatively regulate ROS production, ultimately mitigating I/R‐caused heart injury.

To sum up, we substantiated that F_2_ negatively affects I/R‐induced heart oxidative lesion by activating AMPK‐FoxO1 signalling pathway (Figure 7). Moreover, targeting this antioxidant axis may represent a novel strategy to prevent any other pathologic stimuli‐triggered oxidative injury apart from ischemic heart.

## AUTHOR CONTRIBUTIONS

**Binger Lu:** Formal analysis (equal); methodology (equal); writing – original draft (equal). **Zhuomin Wu:** Formal analysis (equal); methodology (equal); writing – original draft (equal). **Weiliang He:** Methodology (supporting). **Zikai Feng:** Methodology (supporting); writing – original draft (supporting). **Jilin Liao:** Methodology (supporting). **Bin Wang:** Formal analysis (supporting). **Yanmei Zhang:** Formal analysis (supporting). **Fenfei Gao:** Formal analysis (supporting). **Ganggang Shi:** Conceptualization (equal); supervision (equal); writing – review and editing (equal). **Fuchun Zheng:** Conceptualization (equal); supervision (equal); writing – review and editing (equal).

## CONFLICT OF INTEREST STATEMENT

The authors declare no competing interests.

## Supporting information


Data S1:

## Data Availability

All data are available upon request.
