# Supplementary material for: N‐n‐butyl haloperidol iodide mediates cardioprotection via regulating AMPK/FoxO1 signalling
Source: J Cell Mol Med. 2023 Nov 21;28(2):e18049. doi: 10.1111/jcmm.18049 (PMC10826434; doi:10.1111/jcmm.18049)
Supplement: Supplementary file 1 — Data S1: [file JCMM-28-e18049-s001.docx]

**
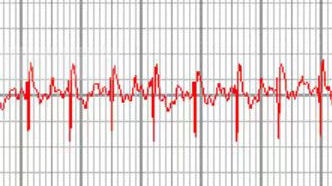

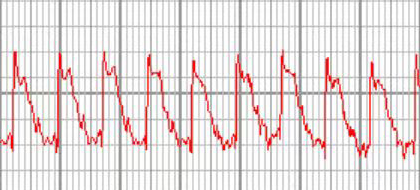
**

**Before ischemia LAD occlusion induced ischemia**

**Supplementary Figure 1. Representative ECG images “before ischemia” and “ischemia”.**

**Supplementary Figure 2. Scheme of the experimental design.**

| Parameters | Sham | I/R | I/R+F_2_ |
| --- | --- | --- | --- |
| HR (bpm) | 482.58 ± 23.81 | 504.15 ± 19.73 | 491.24 ± 31.56 |
| LVESV (μl) | 18.83 ± 1.59 | 33.67 ± 1.94 ^#^ | 25.36 ± 1.61* |
| LVEDV (μl) | 34.78 ± 2.36 | 53.75 ± 2.57 ^#^ | 43.89 ± 3.28* |
| LVAWd (mm) | 1.12 ± 0.08 | 1.10 ± 0.14 | 1.31 ± 0.12 |
| LVPWd (mm) | 1.29 ± 0.17 | 0.84 ± 0.06 ^#^ | 0.96 ± 0.09 |

**Supplementary Table 1. Effects of F_2_ on left ventricular function.** n = 6 mice per group. Data represent mean ± SEM. ^#^*P* < 0.05 vs. the Sham group; **P* < 0.05 vs. the I/R group.

HR, heart rate; LVESV, left ventricle end-systolic volume; LVEDV, left ventricle end-diastolic volume; LVAWd, left ventricle anterior wall thickness at end-diastole; LVPWd, left ventricle posterior wall thickness at end-diastole.
